# Sparse Parallel MRI Based on Accelerated Operator Splitting Schemes

**DOI:** 10.1155/2016/1724630

**Published:** 2016-09-25

**Authors:** Nian Cai, Weisi Xie, Zhenghang Su, Shanshan Wang, Dong Liang

**Affiliations:** ^1^School of Information Engineering, Guangdong University of Technology, Guangzhou 510006, China; ^2^Paul C. Lauterbur Research Centre for Biomedical Imaging, Shenzhen Institutes of Advanced Technology, Shenzhen, China; ^3^Shenzhen Key Laboratory for MRI, Shenzhen, Guangdong, China

## Abstract

Recently, the sparsity which is implicit in MR images has been successfully exploited for fast MR imaging with incomplete acquisitions. In this paper, two novel algorithms are proposed to solve the sparse parallel MR imaging problem, which consists of *l*_1_ regularization and fidelity terms. The two algorithms combine forward-backward operator splitting and Barzilai-Borwein schemes. Theoretically, the presented algorithms overcome the nondifferentiable property in *l*_1_ regularization term. Meanwhile, they are able to treat a general matrix operator that may not be diagonalized by fast Fourier transform and to ensure that a well-conditioned optimization system of equations is simply solved. In addition, we build connections between the proposed algorithms and the state-of-the-art existing methods and prove their convergence with a constant stepsize in Appendix. Numerical results and comparisons with the advanced methods demonstrate the efficiency of proposed algorithms.

## 1. Introduction

Reducing encoding is one of the most important ways for accelerating magnetic resonance imaging (MRI). Partially parallel imaging (PPI) is a widely used reduced-encoding technique in clinic due to many desirable properties such as linear reconstruction, easy use, and *g*-factor for clearly characterizing the noise property [1–6]. Specifically, PPI exploits the sensitivity prior in multichannel acquisitions to take less encodings than the conventional methods [7]. Its acceleration factor is restricted to the number of channels. More and more large coil arrays, such as 32-channel [8–11], 64-channel [12], and even 128-channel [13], have been used for faster imaging. However, the acceleration ability of PPI under the condition of ensuring certain signal noise ratio (SNR) is still limited because the imaging system is highly ill-posed and can enlarge the sampling noise with higher acceleration factor. One solution is to introduce some other prior information as the regularization term into the imaging equation. Sparsity prior, becoming more and more popular due to the emergence of compressed sensing (CS) theory [14–16], has been extensively exploited to reconstruct target image from a small amount of acquisition data (i.e., below the Nyquist sampling rate) in many MRI applications [17–20]. Because PPI and compressed sensing MRI (CSMRI) are based on different ancillary information (sensitivity for the former and sparseness for the latter), it is desirable to combine them for further accelerating the imaging speed.

Recently, SparseSENSE and its equivalence [1, 3, 21–25] have been proposed as a straightforward method to combine PPI and CS. The formulation of this method is similar to that in SparseMRI, except that the Fourier encoding is replaced by the sensitivity encoding (comprising Fourier encoding and sensitivity weighting). Generally, SparseSENSE aims to solve the following optimization problem:

(1) $$\begin{array}{c} \underset{x\in C^{n}}{\min}{\left\|\;Dx\;\right\|}_{1}+\frac{\lambda }{2}{\left\|\;Ax-y\;\right\|}_{2}^{2}, \end{array}$$

where the first term is the regularization term and the second one is the data consistency term. ‖·‖_1_ and ‖·‖_2_ represent separately 1-norm and 2-norm and *x* ∈ *ℂ* ^*n*^ is the to-be-reconstructed image. *D* ∈ *ℂ* ^*m*×*n*^ denotes a special transform (e.g., spatial finite difference and wavelet) and the term ‖*Dx*‖_1_ controls the solution sparsity. *A* and *y* are the encoding matrix and the measured data, respectively:

(2) A=FpS1⋮FpSk∈Ckl×nl<n,y=y1⋮yk∈Ckl,

where *F*_*p*_ is the partial Fourier transform and *S*_*k*_ ∈ *ℂ* ^*n*×*n*^ is the diagonal sensitivity map for receiver *k*. *y*_*k*_ ∈ *ℂ* ^*l*×1^ is the measured *k*-space data at receiver *k*. In this paper, we mainly solve the popular total variation (or its improved version: total generalized variation) based SparseSENSE model, that is, ‖*Dx*‖_1_ = ‖*x*‖_TV_ (or ‖*D*‖_1_ = ‖*x*‖_TGV_).

For the minimization (1), there exists computational challenge not only from the nondifferentiability of *l*_1_ norm term but also from the ill-condition of the large size inversion matrix *A*. Further, the computational complexity becomes more and more huge if we try to improve the performance of SparseSENSE by using large coil arrays, high undersampling factor, or some more powerful transformations (which are usually nonorthogonal) to squeeze sparsity. Therefore, rapid and efficient numerical algorithms are highly desirable, especially for large coil arrays, high undersampling factor, and general sparsifying transform.

Several rapid numerical algorithms can solve the numerical difficulties, which are, for example, alternating direction method of multipliers (ADMM) [26], augmented Lagrangian method (ALM) [27], splitting Bregman algorithm (SBA) [28], splitting Barzilai-Borwein (SBB) [24], and Bregman operator splitting (BOS) [29]. The efficiency of these methods largely depends on the special structure of the matrix operator *D* ^*T*^ *D* (such as Toeplitz matrix and orthogonal matrix) and the encoding kernel (without the sensitivity maps). However, they are not suitable for simultaneously dealing with general regularization operator *D* and the parallel encoding matrix *A*. That is, these algorithms are not able to solve the problem (1) efficiently because the complex inversion of the large size matrix has to be computed, if *D* ^*T*^ *D* and/or *A* ^*T*^ *A* cannot be diagonalized directly by fast Fourier transform (FFT). Alternating minimization (AM) algorithm can address the issue of general *D* and *A*, which is a powerful optimization scheme that breaks a complex problem into simple subproblems [3]. But the addition of new variable may slow the speed of convergence. Our numerical results in Section 4 also demonstrate that the alternating minimization algorithm for large coil data is not very effective in the aspect of convergence speed.  Table 1 illustrates the ability of working on general *D* and *A* (without any preconditioning) for these algorithms. We can see that only AM is able to deal with general operators simultaneously.

To solve the problems existing in the algorithms mentioned above, this paper develops two fast numerical algorithms based on the operator splitting and Barzilai-Borwein techniques. The proposed algorithms can be classified into the forward backward splitting (FBS) method [30] or its variations. Different from some existing fast algorithms, the proposed algorithms can treat general matrix operators *D* and *A* and avoid solving a partial differential equation so as to save huge computational cost. The superiority of our algorithms lies in that operator splitting is applied to both regularization term and data consistency term. Meanwhile, they ensure that a well-posed optimization system of equation is simply solved. Barzilai-Borwein (BB) stepsize selection scheme [31] is adopted for much faster computation speed.

This paper is organized as follows. In Section 2, a review on some related numerical methods for solving the SparseSENSE model is given. In Section 3, two algorithms are proposed as variations of forward-backward splitting scheme. We compare the proposed algorithms with popular algorithms based on the SparseSENSE model in Section 4. In Section 5, we discuss the parameters selection of the proposed algorithms and the connections between the proposed algorithms and the existing algorithms.  Section 6 concludes this paper. Appendix proves the convergence of the proposed algorithms with constant stepsizes.

## 2. Related Work

In the early works, gradient descent methods with explicit or semi-implicit schemes [19, 32] were usually used to solve problem (1), in which the nondifferentiable norm was approximated by a smooth term:

(3) $$\begin{array}{c} {\left\|\;x\;\right\|}_{TV,\varepsilon }=\overset{n}{\underset{i=1}{\sum }}\sqrt{{\left\|D_{i}x\right\|}_{2}^{2}+\varepsilon }, \end{array}$$

where *D*_*i*_ *x* ∈ *ℝ* ^2^ contains the forward finite differences of *x*. The selection of the regulating positive parameter *ε* is crucial for the reconstruction results and convergence speed. A large parameter encourages a fast convergence rate but fails to preserve high quality details. A small one preserves fine structures in the reconstruction at the expense of slow convergence.

The methods in [33, 34] and the split Bregman method [28] equivalent to the alternating direction method of multipliers [26] were presented for solving minimization (1). The efficiency of the algorithms benefits from the soft shrinkage operator and the special structure of the encoding matrix and sparse transform. This requires that both *A* ^*T*^ *A* and *D* ^*T*^ *D* in the optimal equation on *x* can be directly diagonalized by FFT. But these methods may not be suitable for the parallel encoding matrix *A* and more general transform *D*. They are even ill-posed if Null(*A*)∩Null(*D*) ≠ {0}, where Null(·) represents the null space of the operator. In addition, the augmented Lagrangian method in [27] preconditioned the encoding matrix *A* and inevitably computed the inversion of the matrix including general *D* ^*T*^ *D*. So, it is also invalid in the computational efficiency.

The Bregman operator splitting (BOS) method replaces ‖*Ax* − *y*‖_2_^2^ by a proximal-like term [29]. BOS is able to deal with *A* of uncertainty structure by the following iterations:

(4) zk+1=δxk−ATAxk−ysk+1=arg mins⁡s1+ρ2s−wk+Dxk22ρDTD+λδIxk+1=ρDTsk+1−wk+λzk+1wk+1=wk+Dxk+1−sk+1.

However, a partial differential equation including *D* ^*T*^ *D* should be solved as indicated in (4). This equation may bring heavy computation for the general regularization operator *D*. To solve the problem of heavy computation, Ye et al. presented a SBB scheme by utilizing the BB stepsize [24]. However, these algorithms may be not efficient for the general *D* if the matrix operator *D* ^*T*^ *D* cannot be diagonalized by fast transform.

Consequently, minimization (1) can be written as a saddle-point problem:

(5) $$\begin{array}{c} \underset{x\in C^{n}}{\min}\;\underset{w\in X}{\max}\langle \;x,D^{\text{T}}w\;\rangle +\frac{\lambda }{2}{\left\|\;Ax-y\;\right\|}_{2}^{2}, \end{array}$$

where *X* = {*z* : *z* ∈ *ℂ* ^*m*^, |*w*_*j*_| ≤ 1  for  *j* = 1,2,…, *m*}. Although the primal-dual hybrid gradient (PDHG) method alternately updates the primal and dual variables *x* and *z* [35], its efficiency relies on the special structure of *A*. That is, *A* ^*T*^ *A* can be diagonalized directly by fast transform. The alternating minimization (AM) algorithm [3] reduces the PPI reconstruction problem with regularization into the TV-based image denoising and least square (LS) subproblems as

(6) vk+1=arg minvvTV+αv−x22xk+1=arg minxλ2Ax−y22+αv−x22.

The AM algorithm is updated as follows:

(7) zk+1=δkxk−ATAxk−ywk+1=arg minw∈X12w−wk+τkDkvk22vk+1=1+2αθk−1vk+2αθkxk−θkDTwk+1xk+1=λδk+2α−12αvk+1+λzk+1δk+1=Axk+1−xk22xk+1−xk22,

where the stepsize is updated by the rules *τ*_*k*_ = 0.2 + 0.08*k*, *θ*_*k*_ = (0.5 − 5/(15 + *l*))/*τ*_*k*_ [35].

## 3. Algorithm Framework

In this section, we propose two algorithms for solving the SparseSENSE model (1), which are based on the operator splitting scheme and connected by Yosida approximation. We deduce the algorithms with the fixed-point technology as follows.

For the convenience of derivation, we denote ‖*D*·‖_1_ by (*φ*∘*D*)(·) and rewrite the SparseSENSE model as

(8) $$\begin{array}{c} \underset{x\in C^{n}}{\min}\left(\;\varphi \circ D\;\right)\left(\;x\;\right)+\frac{\lambda }{2}{\left\|\;Ax-y\;\right\|}_{2}^{2}. \end{array}$$

By the classic arguments of convex analysis, solution *x* ^*∗*^ to (8) satisfies the first-order optimality condition:

(9) $$\begin{array}{c} 0\in \partial \left(\;\varphi \circ D\;\right)\left(\;x^{*}\;\right)+\lambda A^{T}\left(\;Ax^{*}-y\;\right), \end{array}$$

where ∂(*φ*∘*D*)(*z*) is the subdifferential of (*φ*∘*D*) at point *z*. According to the chain rule, subdifferential ∂(*φ*∘*D*) is identified by

(10) $$\begin{array}{c} \partial \left(\;\varphi \circ D\;\right)=D^{T}\circ \left(\;\partial \varphi \;\right)\circ D. \end{array}$$

By substituting it into (9) and splitting, we get the equivalent formulation:

(11) z∗=x∗−δ−1ATAx∗−y,w∗∈∂φDx∗,0=DTw∗+λδx∗−z∗.

In addition, for any positive number *γ*, we have

(12) w∗∈∂φDx∗⟺γw∗+Dx∗∈γ∂φDx∗+Dx∗⟺Dx∗=Proxγφγw∗+Dx∗⟺γw∗=γw∗+Dx∗−Proxγφγw∗+Dx∗,

where the proximal operator Prox_*γφ*_(*v*) is defined as

(13) $$\begin{array}{c} {Prox}_{\gamma \varphi }\left(\;v\;\right)=\underset{u}{arg\;min}\;\varphi \left(\;u\;\right)+\frac{1}{2\gamma }{\left\|\;u-v\;\right\|}_{2}^{2}. \end{array}$$

Therefore, when the Barzilai-Borwein technique is involved, the solution to the minimization problem (1) can be obtained quickly based on the following updating:

(14) zk+1=xk−δk−1ATAxk−ysk+1=arg mins φs+12γs−γwk+Dxk22γwk+1=γwk+Dxk−sk+1xk+1=zk+1−λδk−1DTwk+1δk+1=Axk+1−xk22xk+1−xk22.

The above iterations can be identified as a forward-backward operator splitting method [36]. Comparing (14) to (4), the partial differential equation in (4) is not involved. Since *φ*(·) = ‖·‖_1_ in the SparseSENSE model (1), the proximal operator is a shrinkage operator that is able to solve *s* ^*k*+1^ quickly from the following formulation:

(15) $$\begin{array}{c} {Prox}_{\gamma {\left\|\cdot \right\|}_{1}}\left(\;v\;\right)=\frac{v}{{\left\|v\right\|}_{2}}\max\left(\;{\left\|\;v\;\right\|}_{2}-\gamma ,0\;\right). \end{array}$$

The proposed algorithm (14) is referred to as the forward-backward operator splitting shrinkage (FBOSS) algorithm.

Considering Moreau's decomposition [30], for the proximal operator there exists a connection as follows:

(16) $$\begin{array}{c} v=Prox_{\gamma \varphi }\left(\;v\;\right)+\gamma Prox_{\varphi^{*}/\gamma }\left(\;\frac{v}{\gamma }\;\right), \end{array}$$

where *φ* ^*∗*^ represents the conjugate function of *φ*. Applying (16) to the updates in (14), for all *k* we get a modified iterating sequences as

(17) zk+1=xk−δk−1ATAxk−ywk+1=arg mint∈X φ∗t+γ2t−wk+γ−1Dxk22xk+1=zk+1−λδk−1DTwk+1δk+1=Axk+1−xk22xk+1−xk22.

Obviously, the proximal operator Prox_*γφ*^*∗*^_ for minimization (1) is a projection operator and *X* = {*x* ∈ *ℂ* ^*n*^ : |*x*_*j*_| ≤ 1 for *j* = 1,2,…, *n*}. According to (15) and (16), we obtain the following computation through a simple derivation:

(18) $$\begin{array}{c} Prox_{{\left\|\cdot \right\|}_{1}^{*}/\gamma }\left(\;\frac{v}{\gamma }\;\right)=\frac{v}{{\left\|v\right\|}_{2}}\min\left(\;\frac{{\left\|v\right\|}_{2}}{\gamma },1\;\right). \end{array}$$

This shows that the modified iteration algorithm (17) is also a fast numerical algorithm for solving minimization (1), which is called the forward-backward operator splitting projection (FBOSP) method. This is because the projection operator keeps the calculation speed same as the shrinkage.

## 4. Numerical Experiments

Three sets of MR data are utilized in the experiments. Data 1 was acquired on a GE 3T scanner (GE Healthcare, Waukesha, WI) with a 32-channel head coil and a 3D T1-weighted spoiled gradient echo sequence (TE = minimum full, TR = 7.5 ms, FOV = 24 × 24 cm, matrix = 256 × 256, and slice thickness = 1.7 mm). Data 2 was of one frame of the dynamic MR data, which was acquired on a 3T Siemens Verio scanner (Siemens Medical Solutions, Erlangen, Germany) (flip angle = 50 degree, TE/TR = 56.6/1.89 ms, Size = 256 × 225 × 17 × 15, FOV = 340 mm × 287 mm, and slice thickness = 6 mm). Data 3 (size = 256 × 256 × 8) was downloaded from the following website: http://www.ece.tamu.edu/~jimji/pulsarweb/ [37].

We can directly reconstruct the sparse data acquired from MRI by means of our proposed schemes. However, we employ the fully acquired data as the reference to make quantitative comparisons between our proposed schemes and the other methods. So, all data sets were fully acquired and then artificially undersampled using nonuniform random sampling masks corresponding to different undersampling factors. Here, a ground truth or reference image *x* ^*∗*^ is set to the square-root of sum of squares of coil images obtained by fully sampled *k*-space data from all channels. Peak signal noise ratio (PSNR) and relative error are employed as the performance metrics, which are defined as 20log_10_⁡(255/‖*x* − *x* ^*∗*^‖_2_^2^) and ‖*x* − *x* ^*∗*^‖_2_/‖*x* ^*∗*^‖_2_, respectively. The sensitivity maps *S*_*j*_ are simulated with the full *k*-space data to avoid the effect of inaccurate sensitivity estimation.

All the comparison algorithms were implemented in MATLAB (version R2013a) and performed on a computer with an Intel(R) Xeon(R) CPU X5690 3.47 GHz, 64 GB of memory, and a Windows operating system. Empirically, we set *λ* = 1.0 × 10^3^ for all algorithms, *ρ* = 0.5 for BOS, and *α* = 1.0 × 10^2^ for AM. For BOS, *δ* = 1. *γ* is set in the range [10^−1^, 10^1^] for FBOSS and FBOSP. Each algorithm is terminated when the relative change ‖*x* ^*k*^ − *x* ^*k*−1^‖_2_/‖*x* ^*k*^‖_2_ reaches the predefined stopping criterion *ε* = 5 × 10^−5^. This criterion could guarantee that the iterative solution for all algorithms approximates to the optimal solution sufficiently.

### 4.1. Comparisons on the TV-Based SparseSENSE Model

In this subsection, the comparisons on the TV-based SparseSENSE model were carried out by BOS [29], AM [3], SBB [24], FBOSS, and FBOSP. We tested on each data set with different undersampling factors.  Table 2 illustrates the numerical comparison results. Here, we did not use 10-fold undersampling factor for data 2 because the reconstruction error for all the algorithms is very large due to high undersampling rate and noise in data acquisition. As shown in Table 2, the proposed algorithms FBOSS and FBOSP achieve better reconstruction performances with less computational time compared to the other three methods.

**Figure 1:**
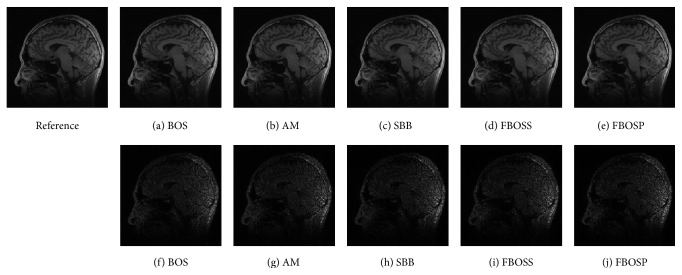
(a)–(e) Reconstructed images with relative error, 3.04%, 2.87%, 2.24%, 2.22%, and 2.23%, separately on data 1 when the undersampling factor equals 10. (f)–(j) Absolute error images corresponding to (a)–(e).

**Figure 2:**
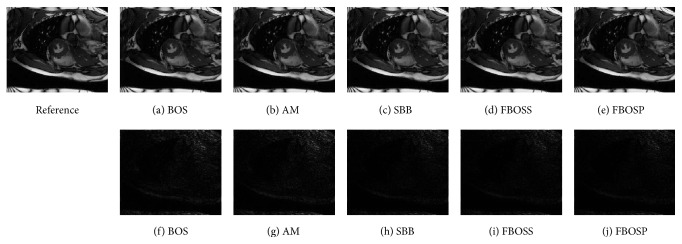
(a)–(e) Reconstructed images with relative error, 1.48%, 1.46%, 1.06%, 1.05%, and 0.97%, separately on data 2 when the undersampling factor equals 6. (f)–(j) Absolute error images corresponding to (a)–(e).

**Figure 3:**
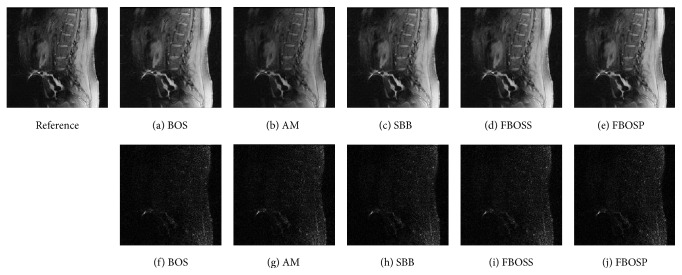
(a)–(e) Reconstructed images with relative error, 6.06%, 6.05%, 5.79%, 5.77%, and 5.73%, separately on data 3 when the undersampling factor equals 10. (f)–(j) Absolute error images corresponding to (a)–(e).

**Figure 4:**
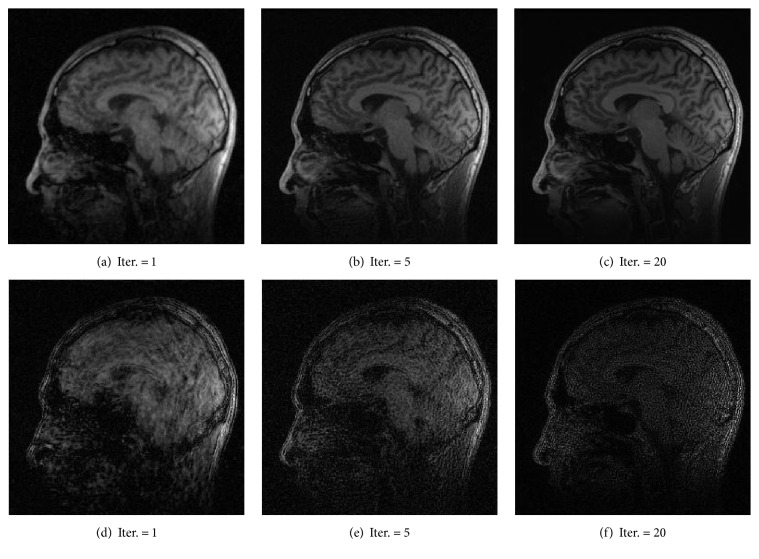
(a)–(c) Reconstruction of data 1 by FBOSS at different iterations when the undersampling factor equals 10. (d)–(f) Absolute error images corresponding to (a)–(c).

**Figure 5:**
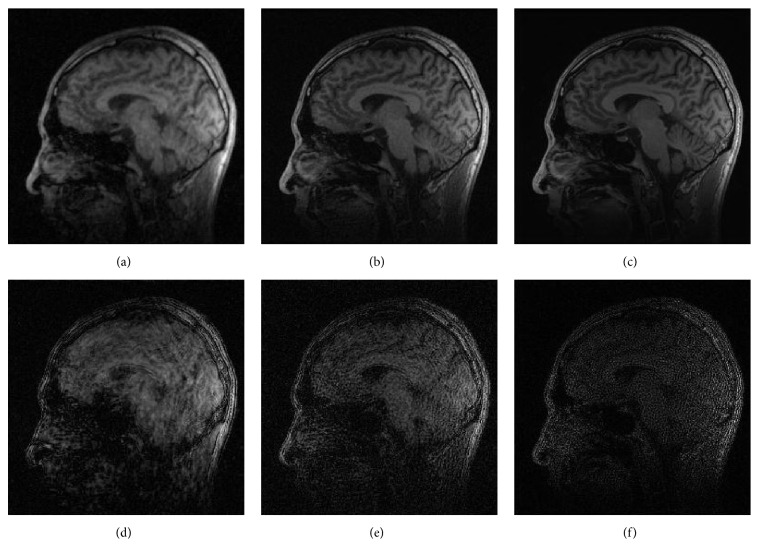
(a)–(c) Reconstruction of data 1 by FBOSP at different iterations when the undersampling factor equals 10. (d)–(f) Absolute error images corresponding to (a)–(c).

As shown in Figures 1, 2, and 3, images reconstructed by the five algorithms have similar reconstruction qualities. In Figures 4 and 5, we give the reconstructed results and error images based on FBOSS and FBOSP at different iterations to illustrate that the proposed algorithms can improve image quality iteratively.

**Figure 6:**
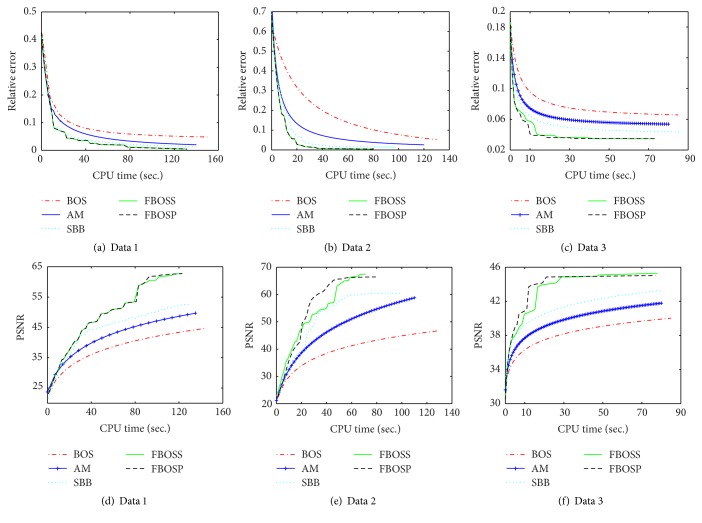
The performance comparisons of the five algorithms for the TV-based SparseSENSE model when the undersampling factors for data 1, data 2, and data 3 are 10, 6, and 10, respectively. (a, b, c) Relative error versus CPU time of data 1, data 2, and data 3. (d, e, f) PSNR versus CPU time of data 1, data 2, and data 3.

Besides, we plot the relative error and PSNR as functions of the CPU time to examine the efficiency of FBOSS, FBOSP compared to BOS, AM, and SBB. As indicated in Figures 6(a), 6(b), and 6(c), the relative error curves of FBOSS and FBOSP descend faster than those of BOS, AM, and SBB. The curve of BOS is far above the others, which implies its lower efficiency. FBOSS and FBOSP have almost identical performance. Figures 6(d), 6(e), and 6(f) demonstrate that PSNR curves of FBOSS and FBOSP have the fastest ascents among these five algorithms.

**Figure 7:**
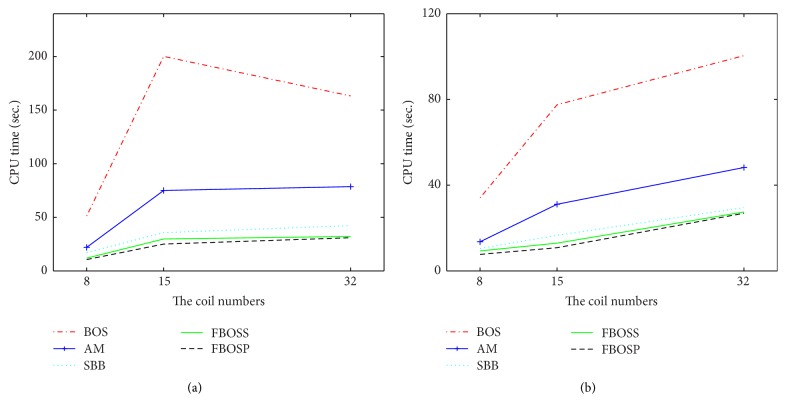
The CPU time of the five algorithms for the TV-based SparseSENSE model as functions of the coil numbers on different undersampling factors. (a) CPU time versus the coil numbers on the 6-fold undersampling factor; (b) CPU time versus the coil numbers on the 4-fold undersampling factor.

The above experiments illustrate that the proposed algorithms FBOSS and FBOSP have better performance than BOS, SBB, and AM in terms of computational efficiency, although *D* ^*T*^ *D* generated from the TV-based SparseSENSE model can be diagonalized by FFT. Besides, as indicated in Table 2, the larger the undersampling factor of the test data, the bigger the CPU time gap between the proposed methods and the others. This gap for the small downsampling factor is not obvious. This is because the initial solution *x* ^0^ for the data with small undersampling factor is close to the optimal solution *x* ^*∗*^; the iterations for the algorithms only take a small amount of time to meet the stopping criterion. Nevertheless, the case for the data with large undersampling factor is opposite. In addition, we find out that the larger the coil numbers of the test data are, the more significant the performances of the proposed methods become. To demonstrate this point, we plotted the CPU time under similar relative error as the function of the coil number of data in Figures 7(a) and 7(b). From the figures we can see that as the coil number increases the proposed algorithms ascend more slowly than the others. The robustness for the coil numbers of data set becomes another advantage of the presented algorithms. Thus, these observations indicate that the proposed algorithms are superior especially in the harsh situation where the data is of large-scale and highly undersampled.

### 4.2. Comparisons on the Second-Order TGV-Based SparseSENSE Model

In this subsection, we only compared FBOSP with AM based on a special second-order TGV reconstruction model to demonstrate the performance of the proposed algorithms for general *D*. This is because BOS and SBB cannot work well for the general sparse transform *D* and FBOSP has an inexact connection to AM.

The second-order total generalized variation [38] is defined as

(19) $$\begin{array}{c} {TVG}_{\alpha }^{2}\left(\;x\;\right)=\sup\left\{\;\overset{}{\underset{\Omega }{\int }}x{\mathrm{div}}^{2}w\;dx,w\in X^{*}\;\right\}, \end{array}$$

where *X* ^*∗*^ = {*w*∣*w* ∈ *C*_*c*_^*k*^(*Ω*, Sym^2^(*ℝ* ^*n*^)), ‖div^*l*^⁡*v*‖_*∞*_ ≤ *a*_*l*_, *l* = 0,1}, *C*_*c*_^*k*^(*Ω*, Sym^2^(*ℝ* ^*n*^)) is the space of compactly supported symmetric tensor field, and Sym^2^(*ℝ* ^*n*^) is the space of symmetric tensors on *ℝ* ^*n*^. By taking *α*_1_ = +*∞*, *α*_0_ = 1, a special form of second-order TGV can be written as

(20) $$\begin{array}{c} {\left\|\;Dx\;\right\|}_{1}={\left\|\;\begin{array}{cc} D_{11}x & \frac{\left(D_{12}x+D_{21}x\right)}{2}\\ \frac{\left(D_{12}x+D_{21}x\right)}{2} & D_{22}x \end{array}\;\right\|}_{1}, \end{array}$$

where *D*_*ij*_  (*i*, *j* = 1,2) is the second-order finite difference and 1 and 2 represent the horizontal and vertical directions, respectively. Obviously, operator *D* in (20) cannot be diagonalized by fast transform. Therefore, BOS and SBB are absent in the comparisons as they are not efficient for the special TGV SparseSENSE model.

**Figure 8:**
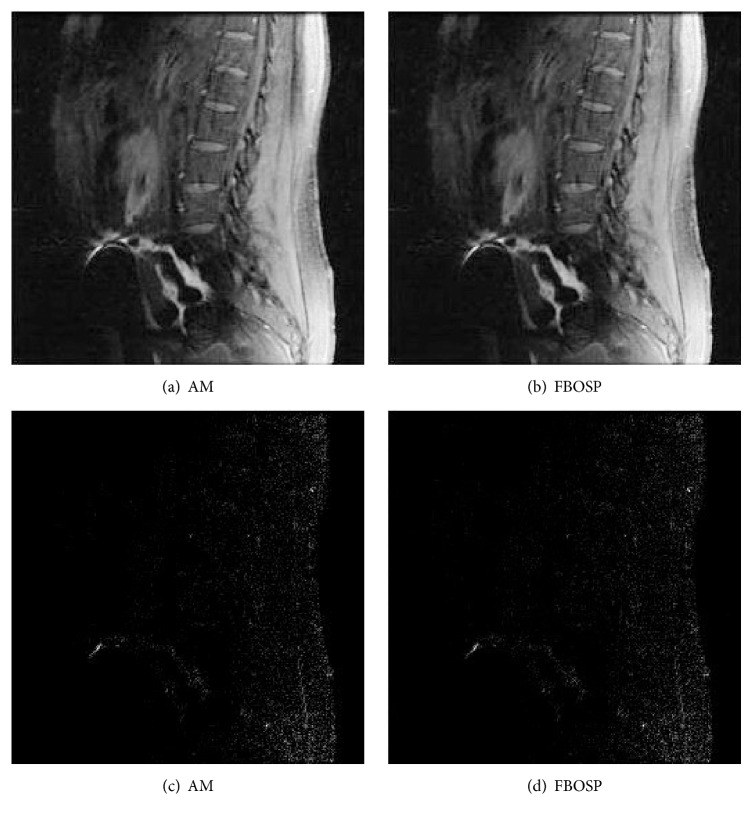
(a)-(b) Reconstructed images with relative error, 1.56% and 1.34%, separately on data 3. (c)-(d) Absolute error images corresponding to (a)-(b).

**Figure 9:**
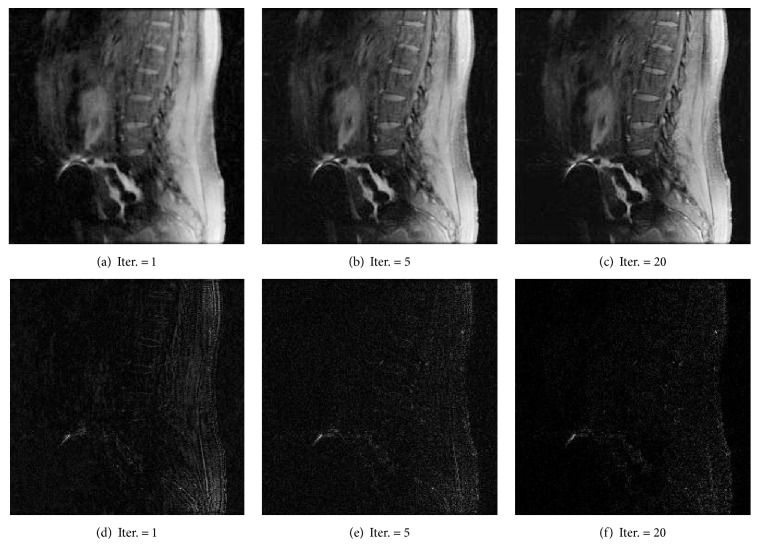
(a, b, c) Reconstruction of data 1 by FBOSP at different iteration. (d, e, f) Absolute error images corresponding to (a, b, c).

**Figure 10:**
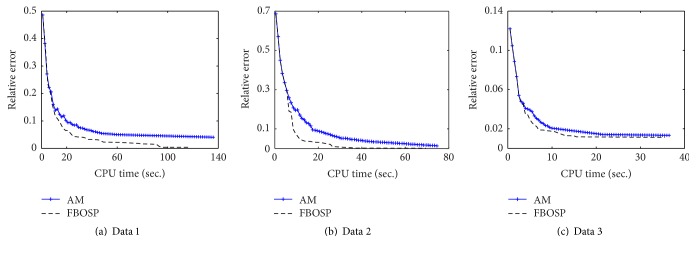
The performance comparisons of AM and FBOSP for the second-order TGV-based SparseSENSE model when the undersampling factors for data 1, data 2, and data 3 are 10, 6, and 4, respectively. (a)–(c) Relative error versus CPU time of data 1, data 2, and data 3.

For the comparisons between FBOSP and AM, we test data 1, data 2, and data 3 corresponding to undersampling factors 10, 6, and 4, respectively.  Table 3 demonstrates the superiority of FBOSP. The reconstructed and error images for AM and FBOSP on data 3 are shown in Figure 8. In addition, we give the reconstructed and error images at different iterations to demonstrate that FBOSP is able to improve image quality iteratively (shown in Figure 9). As shown in Figures 10(a), 10(b), and 10(c), the proposed algorithm FBOSP keeps its superiority in convergence speed.

## 5. Discussions

### 5.1. Parameters Selection

**Figure 11:**
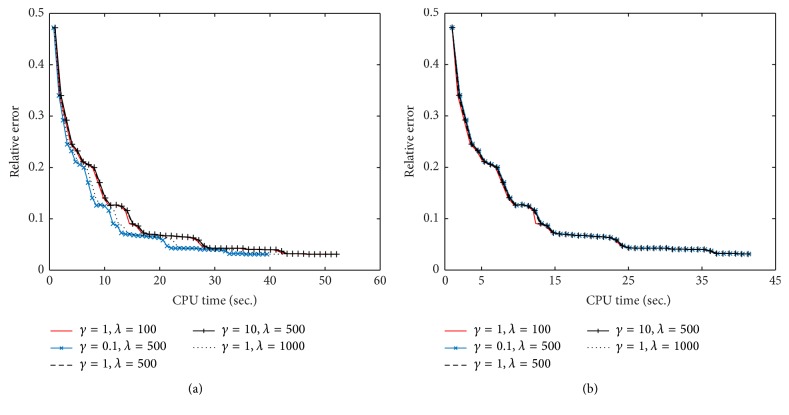
The performance comparisons of different parameters. (a) Relative error versus CPU time on FBOSS; (b) relative error versus CPU time on FBOSP.

In this subsection, we discuss the effect of the involved parameters *γ* and *λ* on the performance of the proposed algorithms for the TV-based SparseSENSE model. Here, data 1 is employed for the test. We plot the relative error as the function of CPU time under the different parameters for FBOSS and FBOSP. As shown in Figure 11(a), FBOSS with smaller *γ* or larger *λ* seems to have better performance than other parameter combinations. The reason is that for a small *γ* the information of gradient image is not easy to lose in the shrinkage threshold procedure. Meanwhile, *x* ^*k*^ can better approximate the optimal solution when *λ* is sufficiently large. In contrast, FBOSP has nearly stable performance under different parameters. Therefore, we selected the parameters of FBOSS for moderated performance and fix the parameters of FBOSP in the experiments.

### 5.2. Connection to the Existing Methods

It is known that the main computational steps of BOS (or SBB) and AM are the splitting Bregman iteration, which are equivalent to ADMM and the PDHG iterations, respectively. In this subsection, we analyze two relations between the proposed algorithms and the existing popular methods: (1) FBOSS and ADMM and (2) FBOSP and PDHG. For simplicity, we simplify (1) by replacing *A* with an identity operator Id.

*(1) Relation between FBOSS and ADMM.* FBOSS can be interpreted as an ADMM method with a semi-implicit scheme applied to the augmented Lagrangian of the original problem as follows:

(21) Lx,s;w=s1−w,s−Dx+β2s−Dx22+λ2x−y22,

where *w* is Lagrangian multiplier. By using ADMM for *L*(*x*; *s*; *w*), the solution to problem (1) (*A* = *I*) can be gained from the following sequence:

(22) sk+1=arg minss1+β2s−Dxk−β−1wk22,wk+1=wk+βDxk−sk+1,xk+1=arg minxβ2sk+1−Dx−β−1wk+122+λ2x−y22.

If *β* = *γ* ^−1^, FBOSS is almost equivalent to ADMM, except that the former employs a semi-implicit scheme for the *x*-subproblem, while the latter employs an implicit scheme. That is why the proposed algorithm performs better than the SBB method although SBB also adopts the BB stepsize scheme.

*(2) Relation between FBOSP and PDHG.* FBOSP based on the projection gradient method can be regarded as a PDHG technique without the proximal term in the gradient descent step, applied to the primal-dual formulation:

(23) $$\begin{array}{c} \underset{x}{min}\;\underset{w\in X^{\prime }}{max}\;\Phi \left(\;x,w\;\right)≔\langle \;x,D^{T}w\;\rangle +\frac{\lambda }{2}{\left\|\;x-y\;\right\|}_{2}^{2}, \end{array}$$

where *w* ∈ *X*′ is dual variable. According to the PDHG iteration, the max-min problem (23) is solved by the update of the sequence:

(24) wk+1=arg maxw∈X Φxk,w−γ2w−wk22,xk+1=arg maxx Φx,wk+1+θ2x−xk22.

By replacing *w* with *t* and taking *θ* = 0 in the above iteration, it is the main procedure of the FBOSP method for minimization (1).

## 6. Conclusions

In this paper, two fast numerical algorithms based on the forward-backward splitting scheme are proposed for solving the SparseSENSE model. Both of them effectively overcome the difficulty brought by the nondifferentiable *l*_1_ norm according to the fine property of proximity operator. They also avoid solving a partial differential equation with huge computation, which has to be solved in the conventional gradient descent methods. The proposed algorithms can treat the general matrix operators *D* ^*T*^ *D* and *A* ^*T*^ *A* that may be not diagonalized by the fast Fourier transform. We compare them with Bregman algorithms [24, 29] and the alternating minimization method [3]. The results show that the proposed algorithms are efficient for solving the SparseSENSE model quickly.

## Figures and Tables

**Algorithm 1:**
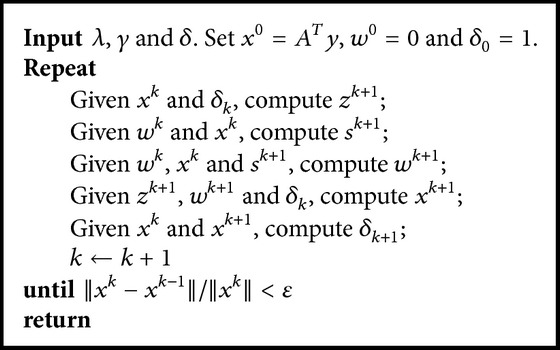
FBOSS for the SparseSENSE model.

**Algorithm 2:**
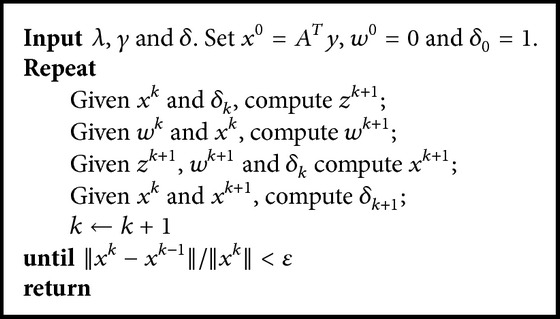
FBOSP for the SparseSENSE model.

**Table 1:** The classification of algorithms for solving the SparseSENSE model.

	ADMM	ALM	SBA	BOS	SBB	AM
Works for general *D*?	No	No	No	No	No	Yes
Works for general *A*?	No	No	No	Yes	Yes	Yes

**Table 2:** Numerical results for the TV-based SparseSENSE model.

	Coil number	Undersampling factor	Performance metric	BOS	AM	SBB	FBOSS	FBOSP
Data 1	32	10	PSNR (dB)	46.87	47.36	49.53	49.61	49.57
			Relative error (*e* − 2)	3.04	2.87	2.24	2.22	2.23
			CPU time (sec.)	325.4	132.3	95.3	55.3	53.0

Data 1	32	6	PSNR (dB)	55.93	56.99	57.62	58.11	57.76
			Relative error (*e* − 3)	10.68	9.49	8.82	8.34	8.68
			CPU time (sec.)	162.7	78.2	45.4	32.2	31.2

Data 1	32	4	PSNR (dB)	61.00	62.41	62.80	63.56	63.56
			Relative error (*e* − 3)	5.98	5.08	4.86	4.45	4.45
			CPU time (sec.)	99.9	48.0	29.3	27.2	26.5

Data 2	15	6	PSNR (dB)	54.22	54.35	57.17	57.25	57.95
			Relative error (*e* − 3)	14.82	14.60	10.55	10.46	9.65
			CPU time (sec.)	199.4	75.1	35.1	29.3	24.7

Data 2	15	4	PSNR (dB)	63.21	65.34	65.93	66.22	66.70
			Relative error (*e* − 3)	5.26	4.12	3.85	3.72	3.50
			CPU time (sec.)	77.7	30.8	16.0	12.7	10.8

Data 3	8	10	PSNR (dB)	40.20	40.21	40.61	40.64	40.68
			Relative error (*e* − 2)	6.06	6.05	5.79	5.77	5.73
			CPU time (sec.)	60.5	24.5	18.0	13.2	10.8

Data 3	8	6	PSNR (dB)	48.86	49.29	50.20	50.13	50.21
			Relative error (*e* − 2)	2.24	2.13	1.92	1.93	1.92
			CPU time (sec.)	51.1	21.4	16.6	11.2	9.8

Data 3	8	4	PSNR (dB)	51.72	51.80	51.78	51.86	51.79
			Relative error (*e* − 2)	1.61	1.60	1.60	1.58	1.60
			CPU time (sec.)	34.0	13.2	10.3	8.5	7.6

**Table 3:** Numerical results for the TGV-based SparseSENSE model.

	Undersampling factor	Performance metric	AM	FBOSP
Data 1	10	PSNR (dB)	47.37	49.62
		Relative error (*e* − 2)	2.87	2.21
		CPU time (sec.)	142.2	60.0

Data 2	6	PSNR (dB)	54.23	55.95
		Relative error (*e* − 2)	1.48	1.22
		CPU time (sec.)	78.4	28.1

Data 3	4	PSNR (dB)	52.01	53.34
		Relative error (*e* − 2)	1.56	1.34
		CPU time (sec.)	19.8	12.0

## Appendix

To illustrate the convergence of the presented algorithms, we show that the proposed algorithm FBOSS with the constant stepsize *δ* converges by utilizing the nonexpansivity of proximity operator in this section. Based on the connection between FBOSS and FBOSP, it is easy to deduce the convergence of FBOSP with the constant stepsize *δ* because the projection operator keeps the contractive property. First, the related theories about the proximity operator are given as follows.

Lemma A.1 A.1. For the given convex function *φ* and its conjugate function *φ* ^*∗*^, it holds for any *a*, *b* ∈ *ℝ* ^*d*^ that

(A.1) Proxγφa−Proxγφb22≤a−b22−I−Proxγφa−I−Proxγφb22.

Next, we give the main conclusion for the convergence of FBOSS with constant *δ* under the nonexpansivity of proximity operator.

Theorem A.2 A.2. Assume *δ* ≥ ‖*A* ^*T*^ *A*‖ ≥ (1/2*λγ*)‖*D* ^*T*^ *D*‖, the sequence {(*s* ^*k*+1^, *w* ^*k*+1^, *x* ^*k*+1^)} generated by Algorithm 1 with constant *δ* converges to the point (*s* ^*∗*^, *w* ^*∗*^, *x* ^*∗*^), where *x* ^*∗*^ is the solution to the SparseSENSE model.

Proof Suppose that the point (*s* ^*∗*^, *w* ^*∗*^, *x* ^*∗*^) satisfies (14), according to (16) and Lemma A.1, we have

(A.2) γwk+1−w∗22=Proxγφ∗γwk+Dxk−Proxγφ∗γw∗+Dx∗22≤γwk−w∗+Dxk−x∗22=γwk−w∗22+2γwk−w∗,Dxk−x∗+Dxk−x∗22.

In addition, by combining the first and the fourth equations in (14), we get

(A.3) 1λγγwk+1−w∗22+xk−x∗H2≤1λγγwk−w∗22+xk−1−x∗H2−xk−1−xkH2−2ATA−1λγDTDxk−x∗,xk−x∗≤1λγγwk−w∗22+xk−1−x∗H2−xk−1−xkH2.

Let (1/*λγ*)‖*γ*(*w* ^*k*+1^ − *w* ^*∗*^)‖_2_^2^ + ‖*x* ^*k*^ − *x* ^*∗*^‖_*H*_^2^ = *M*_*k*+1_, then *M*_1_ ≥ *M*_2_≥⋯ and *M*_*k*+1_ ≥ 0 for all *k*. Hence, lim_*k*→*∞*_⁡*M*_*k*_ exists, which implies that

(A.4) $$\begin{array}{c} \underset{k\to \infty }{lim}{\left\|\;x^{k-1}-x^{k}\;\right\|}_{H}^{2}=0. \end{array}$$

It leads to lim_*k*→*∞*_⁡*x* ^*k*^ = *x* ^*∗*^, which deduces that

(A.5) $$\begin{array}{c} \underset{k\to \infty }{lim}\frac{\gamma }{\lambda }{\left\|\;w^{k+1}-w^{*}\;\right\|}_{2}^{2}=\underset{k\to \infty }{lim}M_{k+1}. \end{array}$$

Moreover, it follows from (14), (16), and Lemma A.1 that

(A.6) Proxγφγwk+Dxk−Proxγφγw∗+Dx∗22≤γwk−w∗+Dxk−x∗22−I−Proxγφγwk+Dxk−I−Proxγφγw∗+Dx∗22=γwk−w∗+Dxk−x∗22−γwk+1−w∗22.

Therefore, by taking limits for both sides of the above inequality, we obtain

(A.7) limk→∞sk+1−s∗2=limk→∞Proxγφγwk+Dxk−Proxγφγw∗+Dx∗2=limk→∞γwk−w∗+Dxk−x∗2=limk→∞γwk−wk+12=0,

which immediately gets

(A.8) limk→∞sk=s∗,limk→∞wk=w∗.

On the other hand, by combining (9), (10), and (12), it is easy to conclude that *x* ^*∗*^ is the solution to the SparseSENSE model (1) if point (*s* ^*∗*^, *w* ^*∗*^, *x* ^*∗*^) satisfies (14).

Through the proof for Theorem A.2, we can immediately obtain the following convergence conclusion for Algorithm 2 with constant *δ*.

Theorem A.3 A.3. Assume *δ* ≥ ‖*A* ^*T*^ *A*‖ ≥ (1/*λγ*)‖*D* ^*T*^ *D*‖, the sequence {(*w* ^*k*+1^, *x* ^*k*+1^)} generated by Algorithm 2 with constant *δ* converges to the point (*w* ^*∗*^, *x* ^*∗*^), where *x* ^*∗*^ is the solution to the SparseSENSE model.
